# An ultrasound-based referential of body height-adjusted normal liver organometry in school children from Bokito in rural Cameroon

**DOI:** 10.1038/s41598-020-59613-z

**Published:** 2020-02-17

**Authors:** Severin Donald Kamdem, Erve Martial Kuemkon, Leonel Meyo Kamguia, Gladys K. Tchanana, Francis Konhawa, Frungwa Nche, Alim Oumarou, Mamadou Hamza, René Ghislain Essomba, Michel Kengne, Bienvenu Etogo Ondigui, Marie Claire Okomo Assoumou, Frank Brombacher, Justin Komguep Nono

**Affiliations:** 1 Division of Immunology, Health Science Faculty, University of Cape Town, Cape Town, South Africa; 2 Cape Town Component, International Centre for Genetic Engineering and Biotechnology, Cape Town, South Africa; 3 Immunology of Infectious Diseases Unit, South African Medical Research Centre, Cape Town, South Africa; 4 School of Health Sciences, Catholic University of Central Africa, Yaoundé, Cameroon; 5 CIAB EXACT Medical Laboratory, Yaoundé, Cameroon; 6 Faculty of medicine and biomedical sciences, University of Yaoundé 1, Yaoundé, Cameroon; 7 Ministry of Public health, Yaoundé, Cameroon; 8 National Public Health Laboratory, Ministry of Public Health, Yaoundé, Cameroon; 9 Wellcome Centre for Infectious Diseases Research in Africa, Institute of Infectious Diseases and Molecular Medicine (IDM), University of Cape Town, Cape Town, South Africa; 10 The Medical Research Centre, Institute of Medical Research and Medicinal Plant Studies, Ministry of Scientific Research and Innovation, Yaoundé, Cameroon

**Keywords:** Parasite host response, Parasitic liver diseases, Paediatric research

## Abstract

The grading system for ultrasonographic assessment of *Schistosoma mansoni* morbidity is crucial for evaluation of control programs. This requires prior definition of normal liver organometric ranges in the population from the endemic area. A cross-sectional study was conducted in a *S. mansoni* endemic area in rural Cameroon. 1002 Participants were screened and 234 of them, free from all common liver-affecting diseases in the area (schistosomiasis, malaria, hepatitis B and C) and with no ultrasonographic signs of liver disease were selected and their liver parameters measured by ultrasonography. All statistics were considered significant for p-values < 0.05. Normal dimensions of livers lobe sizes, portal vein wall thickness and portal vein diameters are reported. The liver organometric data are presented for the entire study population as a whole and separately for males and females as prediction plots, with observed values and fitted regression line with 95% confidence. Reference ranges for liver parameters (size, portal vein thickness and diameter) adjusted for body height established in the current study are novel for Cameroon. The prediction plots generated should improve the accuracy of the assessment of liver morbidity by ultrasonography in the region.

## Introduction

Hepatic schistosomiasis caused by the blood fluke *Schistosoma mansoni* remains one of the most widespread infections worldwide, and in Africa in particular^1^. In endemic areas, the infection is often accompanied by liver and spleen enlargement, which can lead to periportal fibrosis, develop to portal hypertension, oesophageal varices and gastrointestinal bleeding, the main cause of death^2^. The control of the disease and associated sequelae is now increasingly aimed at by national control programmes through regular mass drug administration being provided to school children in endemic areas. The entire success of the global schistosomiasis control program relies on the efficient assessment of these mass drug administration campaigns, particularly in monitoring the ability of these campaigns to mitigate infection and associated morbidity in endemic areas. Although, the monitoring of hepatic schistosomiasis infection by means of accumulating field-deployable diagnostic tools is considerably improving^3^, the infection-associated liver pathology regression/reversal is hard to achieve^3^ and still difficult to be reliably assessed in the field. Egg count in individual stools and/or detection of the parasite antigen in subject body fluids (urine, serum) remain the broadly used field methods for evaluating the effectiveness of anti-schistosomiasis interventions. However, these monitoring methods only reliably provide indication of the infection status but poorly so that of internal pathological changes^3^, given the increasingly acknowledged discrepancies between infection burden and morbidity^4,5^.

Ultrasonography can be used to detect liver enlargement, periportal fibrosis and portal hypertension^3^. The technique has proven to be more reliable than clinical methods for the diagnostic of hepatic pathology^3^. It is a non-invasive, safe, rapid and quite inexpensive technique for evaluating hepatosplenic morbidity due to *S. mansoni* infection^6,7^. This technique allows an evaluation of liver damages, how fast the lesions happen, how far it can be reversed by treatment, and how soon the damages can re-appear following reinfection. Such information are crucial for planning of interventions programmes^3^. To reach a consensus, with the aim to render ultrasonographic assessment of schistosomiasis pathology in studies from different settings comparable by minimizing the intra- and inter-observer variability, WHO heavily advocated for the development of standardized protocols for examination and reporting. The protocol was defined to ensure rapid generation of results comparable from one endemic site to another so as to enable an overall picture on a world-wide scale to help reliably assess the anti-morbidity success of national control programs and ultimately drive a better informed global schistosomiasis control program^3^. For this purpose, an ultrasound-based grading approach for hepatic pathology was defined. Chronologically, a quantitative grading system based on the measurement of the diameter of peripheral portal vein branches, liver and spleen size was first described by the Cairo Working Group^8^ and by the team of Abdel-Wahab^9^. This grading system staged the disease as levels I, II or III with increasing disease severity^10^. Since this scoring system was found difficult to be reliably applied, a qualitative grading system was suggested. For this purpose, other ultrasound meetings were convened in Niamey in 1996 and Belo Horizonte in 1997^11^ where a mix approach combining qualitative and quantitative features was adopted^12^. The latter method combines a qualitative assessment of the liver parenchyma (liver image patterns A to F) an adjustment of organ measurements for height that aimed to reduce the influence of biometric factors discrepancies from one endemic site to another. In addition, since children have been reported to be at higher risk of Schistosomiasis infection^13^, the “Managil classification” has been developed. This latter focused on the thickness and the location of periportal thickening based on the impression of an “experienced ultrasound observer”^14^. It appeared therefore vital for the implementation of this unifying grading approach, a need of standardized organometry^2,11^ which is now widely recognized^8,15^ and recommended by the World health Organisation to regional programs for their surveillance efforts.

Although such referentials are now being defined by measurements of liver lobe size and portal vein wall thickness and diameter adjusted to body height in healthy members in some endemic regions from western Europe, Asia, South America, West African and South African regions^16–20^, no referential is available until date for any country of the Central African Region where hepatic schistosomiasis-driven liver morbidity heavily distributes^4,5^.

The aim of this study was to apply ultrasound liver organometry to healthy schoolchildren from five different villages of a *S. mansoni* endemic area in rural Cameroon, and to establish a referential for the area for liver parameters.

## Results

### Flow diagram of the study

**Figure 1:**
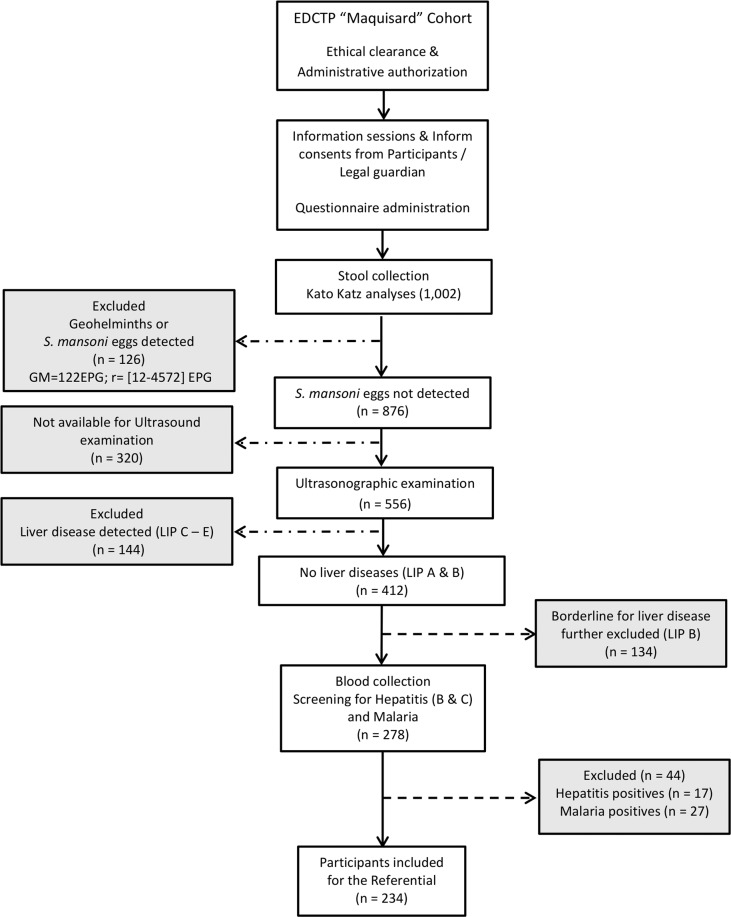
Study flow diagram. The flow diagram shows the selection of participants for the study. This is a sub-study of a bigger ongoing study funded by EDCTP Project TMA 2016 CDF-1571. Filled questionnaire and stool samples were collected from 1,002 participants for Kato Katz analyses. While *S. mansoni* eggs were found in excreta of 126 participants with geometric mean (GM) of 122 Eggs per gram (EPG) of stool for a range = [12–4572] EPG, 876 participants were reported negatives for both schistosomiasis and geohelminths. From the latter group (*S. mansoni* egg-negative participants), ultrasonography was performed in 556 participants. Participants with clearly defined liver disease (Liver Image Patterns C - E) were excluded. Liver Image Pattern B (LIP B) which is borderline was also excluded and only participants with LIP A were included for downstream analyses. For malaria and hepatitis B & C screening, blood was collected from all participants negative for *S. mansoni* eggs with LIPA (n = 278). All malaria and hepatitis B and/or C positive cases where excluded from the study but referred for adequate medical assistance. Finally, 234 participants with LIP A, negative for *S. mansoni* and free of malaria and hepatitis B & C were included for establishment of the liver organometry referential. LIP: Liver image pattern.

Figure 1 shows the strategy of enrollment and selection of participants for the study. This is a sub-study of a bigger ongoing EDCTP-funded study (Project TMA 2016 CDF-1571) on the “Maquisard” cohort. The Maquisard cohort is a cohort of 1002 school-age children in five schools within a radius of 5 kilometers of a schistosomiasis-infested river in the locality of Bokito in rural Cameroon established in 2018, with the aim of identifying host regulators of liver fibrosis in hepatic schistosomiasis-diseased children. The 1002 school children [age range 5–18 years (mean 9.5 years)] were screened as part of the present study through successive steps to exclude schistosomiasis-, geohelminths, malaria- or hepatitis-diseased participants, as indicated in Fig. 1. Following the Kato katz analysis from the 1,002 participants, ultrasound examinations were performed primarily on older and compliant school children (7–18 years). These older children have been previously reported on the site to be more likely to develop liver fibrosis [5]. After further exclusion of all school children with possible liver disease (Liver Image Pattern or LIP B, C, D, E or F), as determined by ultrasonography, 234 participants were defined as healthy controls and included in the determination of the present liver organometric referential.

### Characteristics of the study population

Sociodemographic data of the 234 included school children are summarized in Table 1.

**Table 1:** Age and gender distribution of participants grouped by height.

Body height (cm)	Numbers	Mean age in years (Mode)	Sex ratio (M/F)
[100–120]	10	9.2 (8, 10)	2.3
[121–140]	140	9.8 (10)	0.9
[141–160]	75	12.2 (12)	1.4
>160	09	14.0 (15)	2
Total	234	10.7 (10)	1.1

cm: centimetre; M: Male; F: Female.

The body heights ranged from 111 to 168 cm and were clustered in 4 groups (Table 1), as previously defined in the WHO reference paper [3]. The majority of participants were in the height range of [121–140] cm. Age ranged from 7 to 18 years old with a mean of 10.7 years old and the highest representation at 10 years old. Although the gender repartition was different across the height groups, a sex ratio of 1.1 was reported for the entire study population (n = 234).

### Liver organometry standardised ranges adjusted for body height

**Figure 2:**
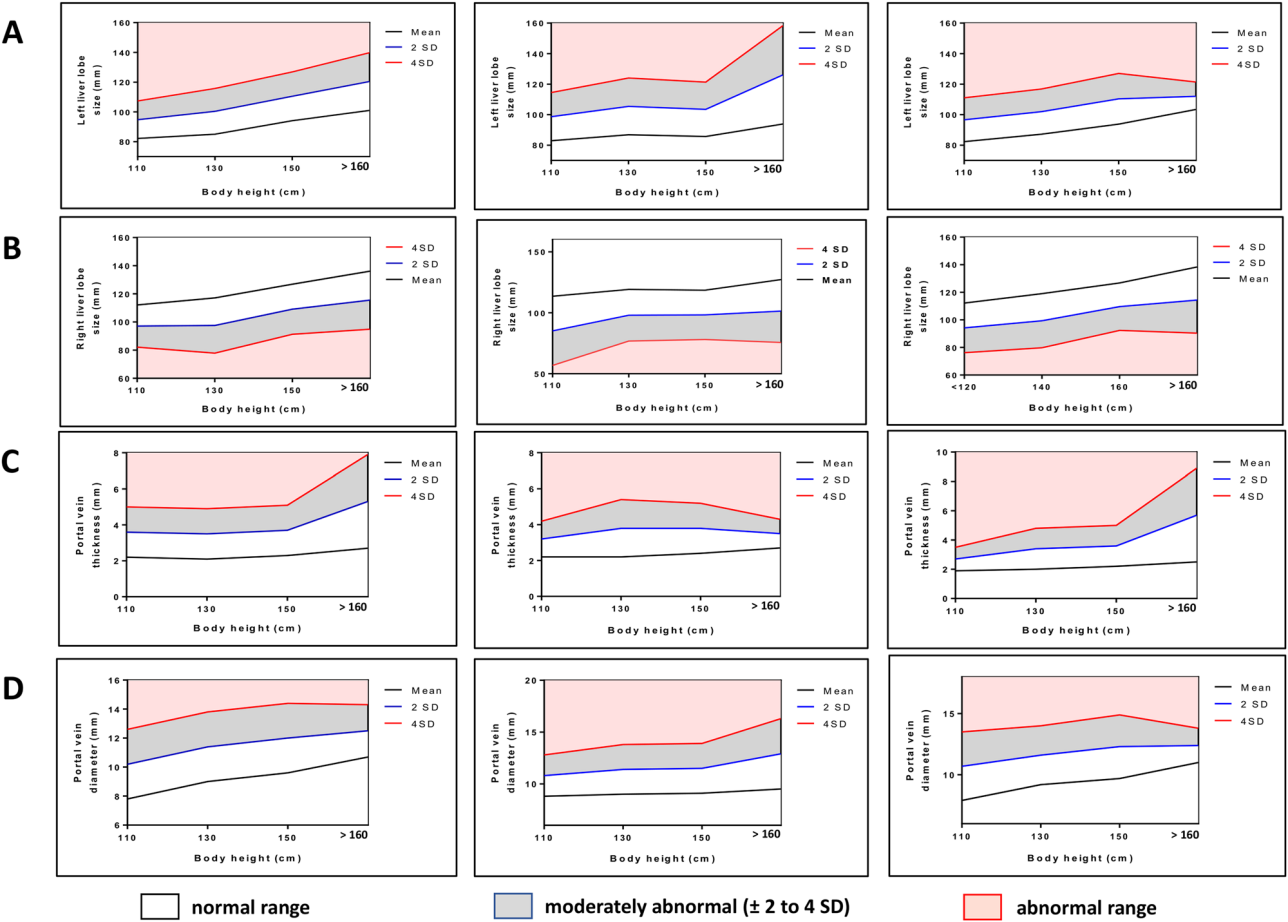
Liver organometry standardised ranges adjusted for body height and by gender. Linear regression was used to define the normal range for liver organometry. (**A**) Left liver lobe size standardised ranges adjusted for body height for the overall study population, females and males. (**B**) Right liver lobe size standardised ranges adjusted for body height for the overall study population, females and males. (**C**) Portal wall thickness standardised ranges adjusted for body height for the overall study population, females and males. (**D**) Main portal vein diameter standardised ranges adjusted for body height for the overall study population, females and males. Normal ranges were defined as the 95^th^ percentiles of the respective variables (mean ± 2 S.D.) (blue line) and values beyond the 95th percentiles were considered abnormal (mean ± 4 S.D.) (red line).

The mean and standard deviation of variation of liver measurements for defined categories of height are shown in Table 2. Observed data and fitted regression lines with 95% confidence and prediction intervals for the individual liver measurements (left liver lobe size, right liver lobe size, portal vein thickness and portal vein diameter) in relation to height are presented in Fig. 2.

**Table 2:** Lliver organometry standardised ranges adjusted for body height and by gender.

Overall participants								
Body height (cm)	Left liver lobe (mm)				Right liver lobe (mm)			
	Mean	SD	2SD	4SD	Mean	SD	2SD	4SD
[100–120]	82.2	6.3	12.6	25.2	112.1	7.5	15.0	30.0
[121–140]	85.0	7.7	15.4	30.8	117.1	9.8	19.6	39.2
[141–160]	94.1	8.2	16.4	32.8	126.9	8.9	17.8	35.6
>160	101.0	9.7	19.4	38.8	136.1	10.3	20.6	41.2
**Body height (cm)**	**Portal branch wall thickness (mm)**				**Main portal vein (mm)**			
	**Mean**	**SD**	**2SD**	**4SD**	**Mean**	**SD**	**2SD**	**4SD**
[100–120]	2.2	0.7	1.4	2.8	7.8	1.2	2.4	4.8
[121–140]	2.1	0.7	1.4	2.8	9.0	1.2	2.4	4.8
[141–160]	2.3	0.7	1.4	2.8	9.6	1.2	2.4	4.8
>60	2.7	1.3	2.6	5.2	10.7	0.9	1.8	3.6
**Females participants**								
**Body height (cm)**	**Left liver lobe (mm)**				**Right liver lobe (mm)**			
	**Mean**	**SD**	**2SD**	**4SD**	**Mean**	**SD**	**2SD**	**4SD**
[100–120]	82.9	7.9	15.8	31.6	113.6	14.2	28.4	56.8
[121–140]	86.8	9.3	18.6	37.2	119.2	10.6	21.2	42.4
[141–160]	85.7	8.9	17.8	35.6	118.5	10.1	20.2	40.4
>60	93.9	16.1	32.2	64.4	127.2	12.9	25.8	51.6
**Body height (cm)**	**Portal branch wall thickness (mm)**				**Main portal vein (mm)**			
	**Mean**	**SD**	**2SD**	**4SD**	**Mean**	**SD**	**2SD**	**4SD**
[100–120]	2.2	0.5	1.0	2.0	8.8	1.0	2.0	4.0
[121–140]	2.2	0.8	1.6	3.2	9.0	1.2	2.4	4.8
[141–160]	2.4	0.7	1.4	2.8	9.1	1.2	2.4	4.8
>60	2.7	0.4	0.8	1.6	9.5	1.7	3.4	6.8
**Males participants**								
**Body height (cm)**	**Left liver lobe (mm)**				**Right liver lobe (mm)**			
	**Mean**	**SD**	**2SD**	**4SD**	**Mean**	**SD**	**2SD**	**4SD**
[100–120]	82.3	7.2	14.4	28.8	112.2	9.0	18.0	36.0
[121–140]	87.2	7.4	14.8	29.6	119.0	9.8	19.6	39.2
[141–160]	93.8	8.3	16.6	33.2	126.8	8.6	17.2	34.4
>60	103.4	4.5	9.0	18.0	138.4	12.0	24.0	48.0
**Body height (cm)**	**Portal branch wall thickness (mm)**				**Main portal vein (mm)**			
	**Mean**	**SD**	**2SD**	**4SD**	**Mean**	**SD**	**2SD**	**4SD**
[100–120]	1.9	0.4	0.8	1.6	7.9	1.4	2.8	5.6
[121–140]	2.0	0.7	1.4	2.8	9.2	1.2	2.4	4.8
[141–160]	2.2	0.7	1.4	2.8	9.7	1.3	2.6	5.2
>60	2.5	1.6	3.2	6.4	11.0	0.7	1.4	2.8

cm: centimetre; mm: millimeter; SD: standard deviation.

To further refine our defined liver organometry referentials and considering the reported gender-based discrepancy between male and female liver organometrics^17^, we separately defined liver parameters distribution by gender (Fig. 2). Individual data sets are also provided to facilitate the use of these refined liver organometric referentials by other studies (Table 2 and Datasets 1 & 2).

Univariate regressions models were designed to further assess the implication of the gender on liver organometrics. In this regard, when considering the equilibrated gender ratio in our total population (1.1), the left liver lobe size (OR: 1.04 95% CI: 1.01–1.07) and the right liver lobe size (OR: 1.03 95% CI: 1.01–1.05) were found significantly bigger in males compared to females.

## Discussion

Using ultrasonography, this survey provides normal, standard organometric data for a Cameroonian population that can be used as the basis for assessing hepatic morbidity in neighbouring *S. mansoni* endemic areas or in other countries with comparable settings (other *S. mansoni* endemic areas either in Cameroon or in the Central Africa). To achieve this objective, with a sample representative of the endemic area, school children were enrolled from 5 neighbouring villages with different *S. mansoni* prevalences.

Prior to selection and inclusion as healthy controls for the establishment of liver parameters normal ranges, participants were screened for *S. mansoni* and/or other geohelminths eggs by the Kato katz technique and only negative cases were retained. However, considering the known low sensitivity of this technique to detect *S. mansoni* and other geohelminths eggs^21,22^, the likelihood of false negative cases for low-burden infections persisted. To render our parasitological screening a bit more robust, 2 stool samples were collected from each participant at 2 different days and examined by 2 different and experienced technicians, as this was reported to improve the Kato-Katz the sensitivity^23–25^. Furthermore, ultrasonographic data (n = 556) were obtained from Kato katz negative-participant and those showing a Liver Image Pattern (LIP) A i.e. with no abnormalities were included for downstream analyses, consistent with the global approval of this pattern as the only pattern of no liver disease in schistosomiasis-affected areas^1,5,26,27^. Of note, LIP B, considered as representative of liver disease by some authors^26,27^ or as borderline by orders^3,5,11^, was also excluded from our study to strictly include participants with no signs of possible liver disease. This caution was additionally taken to further exclude potential kato katz false negative-participants with nascent liver lesions. Moreover, all participants positives for malaria, hepatitis B or hepatitis C were also excluded since all those pathogens develop through a stage within the liver^28,29^, and could possibly lead to abnormal liver organometric parameters^30–32^. This approach had the merit of adding stringency in selecting healthy subjects in our present study when compared to previous studies^16–20^ that have defined regional liver organometric referential in schistosomiasis-endemic areas.

Normal ranges of liver lobe sizes increased with participant height. For instance, in the [100–120 cm] height group, the current reference value of length of the left liver lobe is below 94.8 mm (mean ± 2 S.D.). A size above 94.8 mm is therefore regarded as abnormal considering the traditional cut-off values. Within the (>160 cm) height group, the mean left liver lobe size was 101 mm and a participant with a left liver size of 120 mm is considered as normal. This further confirm the variation of the liver organometry with the body height. Consequently, to avoid misinterpretation and then improve the diagnosis accuracy, our study confirms that, in ultrasound-based hepatic morbidity monitoring studies, each participant should be compared with the standard range of his height group. Our report provides such a standard set of normal ranges of liver organometry adjusted for body height for a population group from Bokito in rural Cameroon.

It should be noted, however, that in as much as we included a variety of sites of the rural locality of Bokito within the Centre region of Cameroon, there is no definitive representation of the entire ecological regions of Cameroon in our sampled study population. The particularity of the study, however, is that school children from Cameroon, a hitherto non-used population pool in African and world schistosomiasis endemic area are now used to define a liver organometry referential. This should be of relatively higher accuracy for studies in the country than foreign referentials. In fact, the absence of any such referential in the central African region proposes the presently defined referential as the closest to reality that investigators and national control programs could use to more reliably assess morbidity in the region.

Comparative analyses of available ultrasound-based liver organometric referentials supports such a claim. When compared to the available referential in Northern Senegal^16^, except for the left liver lobe size, all other liver parameters were higher in participants from Senegal when compared to our Cameroonian cohort. For instance, within the height group (141–160); the right liver love (140.0 vs 126.9); the portal vein thickness (5.0 vs 2.3) and the main portal vein diameter (10.8 vs 9.6) were higher in participants from Senegal compared to our study participants. However, the left liver lobe (84.0 vs 94.1) was smaller in participants from Senegal^16^, though we have to consider that the later study was conducted within the whole population including adult (age range from 4 to more than 40 years) in an area not endemic for schistosomiasis unlike ours. Compared to the referential established in China (age range 4–33 years)^19^, all liver parameters were rather higher in participants from our study population. Within the same height group (141–160), participants from China showed a smaller left liver lobe (63.4 vs 94.1), right liver lobe (111.6 vs 126.9), portal vein thickness (2.2 vs 2.3) and main portal vein diameter (8.6 vs 9.6) compared to our study participants^19^. These observations could be possibly explained by the geographical, nutritional and biometric difference across geographically distinct populations^11^, further confirming the need to establish normal ranges of liver parameters adjusted for body height in as many areas as feasible.

Of note and in contrast to the referential-defining study conducted in Zimbabwe^17^, we found, in our study population, that males displayed a significant bigger left and right liver lobe compared to females. Even though males are generally taller than females, this argument could not be an explanation here given our calculation approach where males and females parameters where only comparatively considered within a similar height range. Certainly, this observation reemphasizes the physiological impact of the gender on the liver organometrics. In fact, univariate regression models further confirmed these discrepancies. This inter-gender heterogeneity in normal liver organometric values might therefore be as important in introducing misinterpretation in studies as that of the erroneous use of a same referential to compare participants from different endemic regions^11^. Our results therefore suggest that despite belonging to a same height group, males and females should have different standardised normal ranges (referential curves) to avoid misinterpretations.

Although increased spleen length and thickness have been reported during *S. japonicum* infection^33^, spleen organometry was not defined in our cohort since spleen enlargement has not been broadly considered as a parameter to assess morbidity owed to *S. mansoni* infection^19^ and consistent with the heavily reported prevalence of malaria, as a spleen-size-altering disease, in the presently studied region of Bokito.

Overall, normal liver organometrics, influenced by biometric factors that reportedly vary across different population and gender groups^11,16^, is hereby defined from school children in a hepatic schistosomiasis endemic site in the subdivision of Bokito in rural Cameroon. A gender-specific liver organometry referential is therefore provided to ameliorate the precision in the ultrasound-based evaluation of liver pathology in the area, complementing currently available referentials from other endemic areas in assisting monitoring surveys of hepatic schistosomiasis-associated liver morbidity in the region.

## Methods

### Ethic statement

Ethical approval was obtained from the Cameroon National Ethics committee for Human Health Research (Approval No. 2018/02/976/CE/NECRHH/SP) followed by authorisations from the Ministry of Basic Education and Ministry of Public Health the of Cameroon (631–12.18). Written informed consents were obtained from all participants and all research was performed in accordance with the Good Clinical Practice (GCP) principles of the International Conference on Harmonization (ICH) and the Declaration of Helsinki. Local authorities and schools directors were also informed and granted us with authorizations. Assisted by school teachers, children and legal guardians were informed of the scope of the study. Written informed consents and assents were given by children and legal guardians. All data were treated anonymously by the research team. All Schoolchildren enrolled were treated with Praziquantel regardless of their parasitological status.

### Study site and participants

The study was carried out in the Bokito subdivision, situated in the Mbam and Inoubou Division, within the Centre region of Cameroon. At around 100 km north of Yaoundé the capital of Cameroon, Bokito is within a transitional zone between forest and savannah. From September to December 2018, data were collected from schoolchildren in five (5) public schools belonging to 5 different villages of the endemic area namely Bongando, Ediolomo, Kedia, Yoro 1 and Yoro 2 public schools. The area is covered by the Cameroon National Program for the Control of Schistosomiasis and Soil Transmitted Helminthiasis, dispensing regularly Albendazole, Ivermectin and Praziquantel. The study took place 5 months after Mass Drug Administration (MDA) of praziquantel within the 5 sites. The protocol of treatment was based on the dose pole strategy, which estimates the number of praziquantel tablets (600 mg each) to be administered from the participant body height^34,35^.

### Data collection

Following the Informed sessions held in presence of schoolchildren and their parents or legal guardians to clearly explain the objective and the methodology of our study, each schoolchild was interviewed by a member of our team, assisted by the parent/legal guardian and the class teacher. All the information was recorded using a questionnaire.

### Parasitological assay

Two stool samples (from 5 days interval) were collected from each participant within a time span of 5 days. Briefly, each schoolchild received a 50 ml pre-labelled and codified screw-cap vials stool container and was requested to provide a fresh morning stool sample. Afterward, within 2 hours, collected stool samples from each of the 5 sites were transported in coolers filled with ice packs to the laboratory of Parasitology of the Medical Research Centre (MRC) of the Ministry of Scientific Research and Innovation (MINRESI) in Yaoundé, Cameroon for examination. From each stool sample, one Kato-Katz thick smear of 41.7 mg (overall 2 smears per participant) was prepared and microscopically examined by two independent technicians to detect and quantify *S. mansoni, S. haematobium* eggs ectopic elimination in stool and other geohelminths as previously described^36^. The geometric mean of intensity (GMI) from the 2 smears was considered as the participant burden.

### Ultrasound examination

All ultrasound examinations were conducted by the same clinician who was unaware of the infection status of the examined participants. Each schoolchild was examined using a portable ultrasonography device with convex transducer of 4 MHz, strictly as per the defined WHO guidelines^3^. Liver lob sizes, portal vein diameter and wall thickness and pathologic liver image patterns were then defined and recorded. Whereas, participants with Liver Image Patterns (LIP) A are not likely to have liver disease^3^ and were therefore considered as negative (controls) for liver disease, participants with LIP ranging from B to F were considered as potential cases of liver abnormalities^11,37^ and excluded.

### Blood collection and assays

Under aseptic conditions, whole blood (4 ml) was collected by a well-trained and authorized phlebotomist into heparin tubes with sodium heparin by venipuncture as previously described^38^. Collected blood samples were stored in coolers filled with ice packs and transported to the laboratory. Once in the laboratory, whole blood smears were prepared, Giemsa-stained and analysed by microscopy as described by^39^ for malaria parasites detection. Subsequently, plasma samples were prepared by centrifugation and stored at −80 °C until use. Hepatitis B and C diagnostics were performed using respectively DiaSpot HBsAg and DiaSpot HCV Ab test strip from DIASPOT^TM^, Indonesia. All the assays were performed following the instructions from the manufacturers.

### Statistical analysis

Data collected were first entered into an excel sheet and statistical analyses were conducted using R studio software and GraphPad Prism 6 (http://www.prism-software.com). Descriptive measures (means and standard deviation) were used to summarize data. Graphs were plotted using GraphPad Prism 6. Furthermore, univariate logistic regression was used to assess the influence of participant gender on liver parameters sizes. Using linear regression, normal ranges were defined as the 95^th^ percentiles of the respective variables (mean ± 2 S.D.) and values beyond the 95th percentiles were considered abnormal. For all analyses, a *p*-value < 0.05 was considered statistically significant.

## Supplementary information

Dataset 1.

Dataset 2.

## Data Availability

All data are available within the present manuscript and Supplementary Information.
